# Phylogeography of a Marine Insular Endemic in the Atlantic Macaronesia: The Azorean Barnacle, *Megabalanus azoricus* (Pilsbry, 1916)

**DOI:** 10.1371/journal.pone.0124707

**Published:** 2015-04-28

**Authors:** Javier Quinteiro, Pablo Manent, Lois Pérez-Diéguez, José A. González, Corrine Almeida, Evandro Lopes, Ricardo Araújo, Gilberto P. Carreira, Manuel Rey-Méndez, Nieves González-Henríquez

**Affiliations:** 1 Molecular Systematics Laboratory, Department of Biochemistry and Molecular Biology, University Santiago de Compostela, A Coruña, Galicia, Spain; 2 Departament of Biology, University of Las Palmas de Gran Canaria, Las Palmas de Gran Canaria, Spain; 3 Departament of Enginery and Sea Sciences. University of Cabo Verde, Mindelo, São Vicente, Cabo Verde; 4 Natural History Museum of Funchal, Funchal, Madeira, Portugal; 5 Regional Directorate of Sea Affaires, Regional Secretary of Natural Resources, Horta, Açores, Portugal; Central Michigan University, UNITED STATES

## Abstract

The Azorean barnacle, *Megabalanus azoricus* (Pilsbry, 1916), is a Macaronesian endemic whose obscure taxonomy and the unknown relationships among forms inhabiting isolated Northern Atlantic oceanic islands is investigated by means of molecular analysis herein. Mitochondrial data from the 16S rRNA and COX1 genes support its current species status, tropical ancestry, and the taxonomic homogeneity throughout its distribution range. In contrast, at the intraspecific level and based on control region sequences, we detected an overall low level of genetic diversity and three divergent lineages. The haplogroups α and γ were sampled in the Azores, Madeira, Canary, and Cabo Verde archipelagos; whereas haplogroup β was absent from Cabo Verde. Consequently, population analysis suggested a differentiation of the Cabo Verde population with respect to the genetically homogenous northern archipelagos generated by current oceanographic barriers. Furthermore, haplogroup α, β, and γ demographic expansions occurred during the interglacial periods MIS5 (130 Kya - thousands years ago -), MIS3 (60 Kya), and MIS7 (240 Kya), respectively. The evolutionary origin of these lineages is related to its survival in the stable southern refugia and its demographic expansion dynamics are associated with the glacial-interglacial cycles. This phylogeographic pattern suggests the occurrence of genetic discontinuity informative to the delimitation of an informally defined biogeographic entity, Macaronesia, and its generation by processes that delineate genetic diversity of marine taxa in this area.

## Introduction

The Azorean barnacle belongs to the taxonomically problematic genus *Megabalanus* [1]. In the latest major revision of the genus it was classified as *Megabalanus azoricus* (Pilsbry, 1916) [2]. It was formerly included in Darwin’s [3] subdivision A of the genus *Balanus*, which contained the larger forms of Balani, as a variety of *Balanus tintinnabulum* (Linnaeus). Hoek [4] proposed the term Megabalanus for these forms and Pilsbry [5] used it as a subgenus. Newman and Ross [1] elevated it to the generic rank, *Megabalanus*, which currently includes 23 species [2]. Recently, it has been referred to as *Megabalanus tintinnabulum* [6]. In some instances, it is cited as *M*. *azoricus* when located in the Azores and as *M*. *tintinnabulum* when found in the other Macaronesian archipelagos [7]. Although a recent morphological re-examination clearly described *M*. *azoricus* from the Azores [8], their taxonomic status and phylogenetic relationships with the other Macaronesian forms remains unclear [9]. It is apparently endemic to the Macaronesian archipelagos, although some records suggest the presence of similar forms as far south as the St. Helena Islands [2]. Additionally, their fouling ability confers a high dispersion capacity, which may have altered their original distribution [9]. This occurs in several *Megabalanus* species, including *M*. *coccopoma*, *M*. *rosa*, and *M*. *volcano*, whose current cosmopolitan distributions are associated with fouling in ocean-crossing vessels [10].

*Megabalanus azoricus*, common, native endemic form in the Azores islands, has been reported from different collections [9]. Historically, the species is a highly appreciated seafood and is consumed locally [6]. Topotypes are well characterized [8] and their reproductive biology described [11]. Darwin [3] assigned the Madeira islands *Megabalanus* specimens to the *M*. *tintinnabulum* taxa. A re-examination of these specimens assigned them to the *M*. *azoricus* taxa [12]. González et al. [13] reported the occurrence of *M*. *azoricus* in the Canaries, tracing archeological remains of the species back to 800 years ago. Although present throughout the archipelago, it is more abundant in the eastern islands, closest to the African coast [13]. Although *M*. *azoricus* has been cited for the Cabo Verde Islands, were it is also consumed as a seafood, there is no detailed study of this population [2, 14].

The absence of genetic data for *M*. *azoricus* means that morphological characters have been exclusively used for species diagnosis to date. The wall plates, terga, and scuta have been well described [2] and facilitate differentiation from other co-generic species. Recently, a re-examination of Azorean specimens confirmed the validity of these external characters, the thoracic and mouth appendages were also used [8]. However, genetic data is necessary to confirm the expected congruence of genetics with morphology, providing a standard barcode for species identification, to identify taxonomic distinctiveness and relationships with co-generic species, and to define the homogeneity or partition of genetic diversity along the Macaronesian geographic range under discussion. The resulting species delimitation is a fundamental prerequisite for biogeographical hypothesis testing [15]. Available mitochondrial DNA data for co-generic species available include partial COX1 and 16SrDNA sequences and the complete mitochondrial genome for *M*. *volcano* (NC_006293). Homologous data has been used in similar biological and biogeographic studies to explore taxonomic and phylogeographic issues. Consequently, the highly complex taxonomic ranks of barnacles have been phylogenetically evaluated [16–19]. However, there are also cases where genetic analysis has assisted low-level taxonomic revisions. For example, the naked barnacle, *Heteralepas japonica*, located in Philippine waters, shows a distinctive clade divergent from the Japanese and Taiwanese populations and is now a new species, *Heteralepas cantelli* [20]. Similar work contributed to new species descriptions in the *Tretaclita squamosa* complex in Asia [21, 22]. Analysis of genetic diversity through the East Atlantic distribution range of *Pollicipes pollicipes* detected a distinctive, monophyletic population in Cabo Verde [23], which was subsequently described as a new species [24]. Phylogeographical inferences include the relationship between local oceanography and clade structure in the morphologically homogeneous acorn barnacle, *Chthamalus malayensis*, along the Indo-West Pacific [25]. The effect of historic glacial events on the genetic diversity of European and North American populations of *Semibalanus balanoides* was investigated by analyzing control region variation [26]. The mechanisms delineating the range distribution in *Tetraclita rubescens*, focusing on migration load and current climate change effects, have also been investigated [27].

A prerequisite for Macaronesian endemism is the existence of a well-defined biographic entity supporting it. Throughout the last 60 My (million years) the Macaronesian region has been evolving geologically, and at present is comprised of diverse seamounts and the Azores, Madeira, Canaries, and Cabo Verde archipelagos [28]. However, their biogeographic delimitation has also been evolving and is mainly inferred from vegetation [29] but also from ichthyological distribution data [30], from a nuclear biogeographic unit including the volcanic provinces of the Canaries and Madeira [31], the Azores archipelago [32], the Cabo Verde Islands [33], and frequently incorporating the Iberian and African continental coastal areas [34]. As a result, Macaronesia spans an extensive latitudinal range from 40–15° N, a climate gradient from temperate, Mediterranean, and tropical oceanic climate regimes, a gradient of distances to the mainland, and diverse floristic affinity patterns. This all raises the question of whether or not Macaronesia as a biogeographic entity is a valid zoogeographical concept [35].

Some crucial features of the *M*. *azoricus* species are digonic hermaphroditism in sessile adults, development of planktotrophic larvae, dependence on exposed rocky shores in the intertidal habitat, and insular distribution restricted to the northeastern Atlantic oceanic islands [11]. These Macaronesian islands are a source of evolutionary paradigms [35]. They emerged in the open-ocean where contrasting hydrographic mechanisms that favors either dispersion or isolation of marine organisms occurs [23, 36, 37]. Sessile intertidal organisms, such as barnacles, show a hydrographic-dependent mobility throughout the planktotrophic larval phase. The genetic signature expected from this dispersal pattern can be recovered after discriminating it from signals produced by a number of species-specific processes and factors delineating genetic diversity within a marine species, including life history traits, behavior, selection, and historical demography [37]. Therefore, the delineation of intraspecific genetic diversity in this barnacle species must match the eco-geographical marine continuum expected for the insular Macaronesia biogeographical region.

The high level of direct exploitation as a food source has been pinpointed as the cause of declining populations in the Azores archipelago [6, 11], a situation that can likely be extrapolated to the rest of Macaronesia. Accordingly, based on the “global/regional importance, rarity, sensibility, keystone status, decline, and threat”, *M*. *azoricus* has been placed on the OSPAR 2010 list of threatened and/or declining species and habitats [38]. In this critical context, taxonomic, genetic, and phylogeographic data will make a valuable contribution to resource management and conservation strategies of the Azorean barnacle [37].

The present work includes the characterization of diverse conserved codifying and non-codifying hypervariable mitochondrial sequences to allow both taxonomic and population structure analysis. Thus, the phylogenetic and descriptive analysis of the intraspecific genetic diversity in *M*. *azoricus* allow us to evaluate hypotheses on their taxonomic and endemic status throughout their Macaronesian geographic range. Subsequently, inferences on gene flow and historical demography facilitate the definition of a specific phylogeographic pattern consistent with the hypothesis of a putative Macaronesian biogeographic marine entity, and with the local hydrography and paleoclimatology processes.

## Materials and Methods

### Sampling and DNA isolation

**Fig 1:**
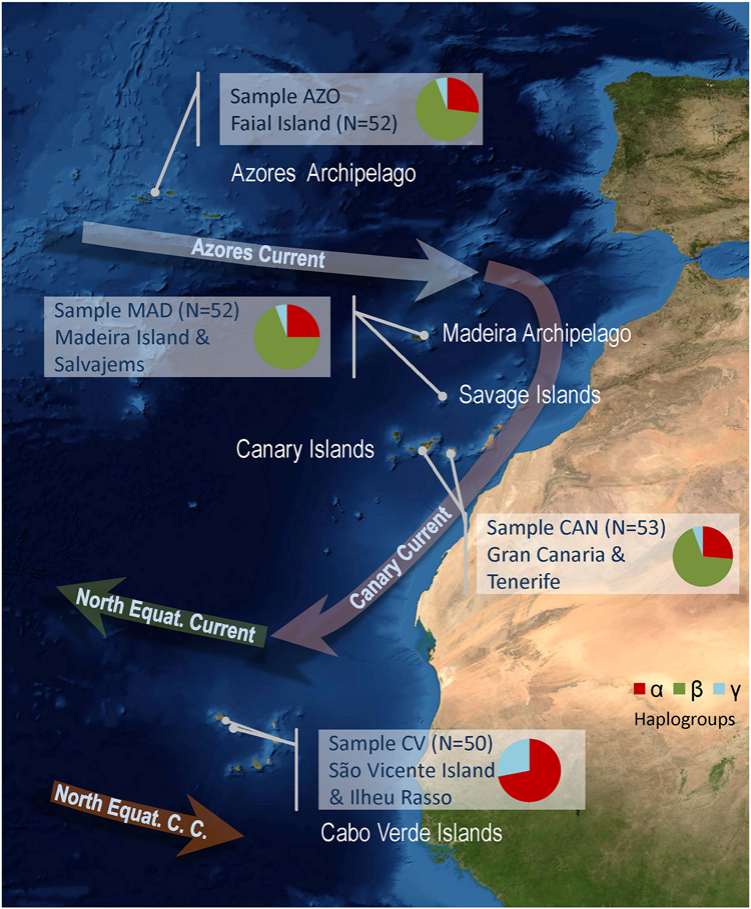
Sampling locations and haplogroup frequencies. Image (NASA) of the north-eastern Atlantic area indicating the sampling locations for each of the Macaronesian archipelagos. Arrows indicate the main surface currents and their directions. Haplogroup (α, β, and γ) frequencies estimated for each sampling site are presented in pie charts.

Four *Megabalanus azoricus* (Pilsbry, 1916) samples (*N* ≥ 50 individuals) were collected from each of the Macaronesian archipelagos, including the Azores, Madeira (including Savage), Canaries, and Cabo Verde Islands (Fig 1, Table 1), comprising a total of 207 specimens. No regulations were adopted in response to the suspected declining status [38]; thus, no specific permissions were required at the sampling locations during the collection period. Therefore, the work did not involve endangered or protected species. However, at present, capture is formally prohibited at the Canary Islands to stock recovery. Immediately after collection, the complete individuals were fixed in 70% ethanol and preserved at −20°C until analysis. A specimen of the sympatric species *Megabalanus tulipiformis* (J. González, ICCM Collection: ICCM21A-SP370) was also collected in the Canaries. A muscle tissue section was excised to obtain a 30 mg tissue sample for total DNA isolation with the E.Z.N.A.® Tissue mollusk kit (Omega Bio-Tek, Norcross, GA). DNA was spectrophotometrically quantified with SmartSpec Plus (Bio Rad, Hercules, CA) and its integrity was then evaluated by 1.5% agarose gel electrophoresis. The BANGEMAC Network (http://bangen-pct.org/) maintains a collection of the analyzed tissues and DNA samples.

**Table 1:** Genetic diversity values, mismatch distribution parameters, neutrality, and demographic expansion test for the *Megabalanus azoricus* populations sampled and haplogroups detected.

Sampling area	Location	*N*	Date	N hap.	*h*	π	Tajima's *D*	Fu's Fs	*τ*	Theta (θ) 0	Theta (θ) 1	H's R i	SSD	*R*_2_
Azores	Faial	52	07/01/2011	27	0.8967	0.012416	−0.92625	−12.89651*	9.834	0	6.612	0.03415	0.02682	0.0705
Madeira	Madeira	45	14/10/2011	34	0.9630	0.013201	−1.17773	−22.87951*	0.811	0	9999	0.04911	0.27230	0.0633
	Salvagems	7	24/03/2010											
Canaries	Tenerife	33	04/05/2010	34	0.9412	0.013988	−1.26907	−21.21641*	10.086	0	8.943	0.01269	0.01128	0.0553*
	Gran Canaria	20	13/04/2011											
Cabo Verde	Ilhéu dos Pássaros, Sao Vicente	22	21/12/2010	48	0.9984	0.021151	−1.81523*	−24.68571*	5.635	5.525	136.719	0.00413	0.00623	0.0434*
	Ilhéu Rasso	28	05/07/2012											
Haplogroup α		77		60	0.9850	0.010734	−2.34008*	−25.68379*	3.316	1.781	33.145	0.00895	0.00012	0.0241*
Haplogroup β		107		62	0.9607	0.004718	−2.58151*	−27.40727*	1.965	0	99999	0.05440	0.00119	0.0153*
Haplogroup γ		23		21	0.9921	0.015736	−1.25601	−13.9761	6.874	0.004	56.367	0.01350	0.00363	0.0765*
*Megabalanus azoricus*		207		133	0.9649	0.018129	−1.78818*	−24.483*	11.125	0	14.073	0.00823	0.00713	

*Significant values

The complete individuals and the external and internal views of the scuta and terga plates were observed to verify their congruence with the morphological descriptions available for *M*. *azoricus* [2, 8].

### Sequence amplification and sequencing

The intraspecific analysis was carried out with the complete mitochondrial control region sequences flanked by the 12S rRNA and tRNA-Ile genes. Moreover, the partial mitochondrial 16S rRNA and COX1 gene sequences were obtained in a subset of the sample for the interspecific comparative analysis of sequences in phylogenetic reconstruction and DNA barcoding. The near complete mitochondrial 16S rRNA was amplified with Cirr-16S-5F (5′-CAACCTGGCTYACGCCGGTCT-3′) and Cirr-Val-3R (5′-GCTGTTTAAGCATTTCATTTACACTGAAAAGG-3′) primers. The fragment in the COX1 mitochondrial gene was amplified using the LCO1490 and HCO2198 primers [39]. Finally, amplification of a fragment containing the complete mitochondrial control region and flanking sequences was carried out with the primers Mazo-12S-1F (5′-CTATCAAAGTAATCCTTTTGTCAGGCA-3′) and Mazo-Ile-2R (5′-′GATAACAACACGGACCTCAACGAT-3′).

Amplifications were performed in a 15 μL reaction volume using GeneAmp 9700 thermocyclers (Applied Biosystems, Carlsbad, CA). PCR reactions comprised 1X AmpliTaq 360 buffer (Applied Biosystems), 3.5mM MgCl_2_, 200 μM each dNTP, 0.13 μM each primer, 0.15 units of AmpliTaq 360 (Applied Biosystems), and 10–50 ng of total DNA. However, amplification within the COX1 gene was performed with 2.5mM MgCl_2_. The consensus PCR profile was 94°C for 3 min, followed by 36 cycles of 94°C for 30 s, 50°C for 30 s, and 72°C for 60s, then 72°C for 7 min.

The PCR products were digested with Exo-SAP-It (Affimetrix, Santa Clara, CA), to remove primers and to deactivate unused dNTPs, and sequenced in both directions using the BigDye 3.1 sequencing kit (Applied Biosystems). The extension products were purified with DyeEx columns (Qiagen, Hilden, Germany) and separated and detected in a 3730xl Genetic Analyzer (Applied Biosystems). Following chromatogram revision and trimming with Sequence Scanner software (Applied Biosystems), the sequences were aligned with ClustalX [40] implemented in BioEdit [41].

### Taxonomic and phylogenetic inference

The external morphology of the specimens was verified for compliance with previous descriptions of the Azorean barnacle [1, 2, 8]. A meta-analysis of the phylogenetic relationships among *Megabalanus* taxa based on the available homologous 16S rRNA partial gene data was performed to corroborate the phylogenetic singularity of the sampled *M*. *azoricus* and define their phylogenetic relationships in a limited but informative data set. Additionally, our dataset (N = 54) was compared with the available *Megabalanus* COX1 sequences through BLAST (http://blast.ncbi.nlm.nih.gov/) and BOLD [42] to find homologous sequences with maximum similarity. The ribosomal phylogenetic inference was based in the Bayesian criterion in MrBayes v.3.2 [43]. The HKY model [44] was selected for 16S rRNA alignment based on AIC estimations in jMoldeltest v.2.1.1 [45] and implemented in MrBayes with an α parameter of the γ distribution = 0.32 and the proportion of invariable sites = 0.57 sets as priors. Markov chain Monte Carlo (MCMC) analysis was run twice for 10,000,000 generations with default parameters being obtained the 50% majority rule tree.

### Population structure analysis

Genetic diversity estimations for the control region and COX1 sequences were obtained in Arlequin v. 3.5 [46] and DnaSPv.5.10 [47], these included nucleotide diversity (π), haplotype diversity (*h*), and Tajima’s *D* in populations and observed haplogroups.

The demographic expansion hypothesis within this species was tested with alternative tests based on different criteria. First, using the distribution of the observed number of differences between sequence pairs obtained with Arlequin v3.5 [46], the so called mismatch distributions, which are usually unimodal in populations under recent demographic expansion [48, 49]. The mismatch distributions were obtained for both populations and haplogroups. The adequacy of the expansion model was assessed using a parametric bootstrap approach [50], by means of i) the sum of squared deviations (SSD) between the observed and expected distributions, and ii) computing the raggedness index (H’s Ri) from the observed distribution as suggested by Harpending [51]. The corresponding estimators of time of expansion (*τ*) and mutational parameters *θ*_0_ and *θ*_1_, before and after the expansion event, were also obtained in Arlequin by parametric bootstrapping [50].

We also performed the Tajima’s [52] and the Fu’s [53] tests for neutrality. Moreover, the significant values for the Tajima’s D statistic can be related to demographic dynamics [54] and the Fu’s *Fs* statistic shows large negative values when estimated from a population undergoing demographic expansion [53]. For small sample sizes the *R*_2_ test is superior in detecting population growth [55].

The pairwise *F*_*ST*_ among the four sampled populations and their significance were computed in Arlequin v.3.5 [46]. The significance of the partition of genetic diversity within a jerarquized structure including populations and their groups was tested with AMOVA, also in Arlequin v.3.5 [56]. The population size dynamics over time were reconstructed by Bayesian coalescent inference [57]. A Bayesian skyline plot of effective breeding population size (*N*_e_) through time was obtained with BEAST (version 1.7.5) [58] under the HKY model [44] with site rate heterogeneity, specifying an estimated fixed mean substitution rate value, using a linear model, and 50,000,000 generations for the MCMC algorithm.

Relationships among the sampled haplotypes, their relative frequencies, and geographic locations were graphically described by means of a median-joining network [59] in Network v.4.6 (Fluxus Technology Ltd., Suffolk, UK). Additionally, the phylogenetic relationships and assemblage of haplotypes were corroborated in a neighbor-joining optimal mid-point rooted tree inferred with MEGA 5 [60], with the branch support estimated by bootstrapping (2,000 replicates). Considering the available models of sequence evolution for Arlequin and MEGA calculations, the model that best fit the control region sequence data based on AIC estimations from jMoldeltest v2.1.1 [45] was the Tamura-Nei [61], with an α value = 0.2220 for the gamma distribution.

## Results

### Species identification

**Fig 2:**
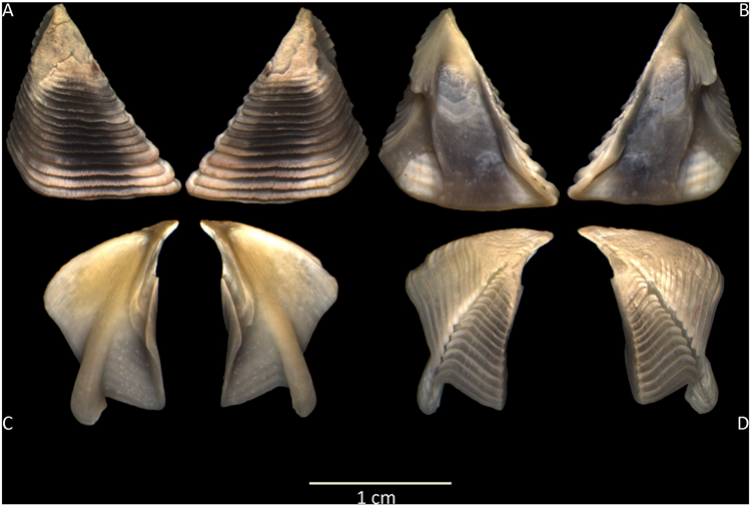
View of terga. Exterior (A) and interior (B) views of scuta and exterior (D) and interior (C) view of terga from a *Megabalanus azoricus* individual sampled in the Azorean archipelago (Terceira Island).

Major revisions of the complex *Megabalanus* taxonomy include *Megabalanus azoricus* at the species rank and define their external diagnostic characters [1, 2]. The individuals sampled throughout the Macaronesian archipelagos, including the Azores type location (Terceira, Azores), exhibit morphological characters that agree with the available descriptive data for *M*. *azoricus* [2]. Thus, the parietal plates are predominantly ribbed. The straight scutum has prominent growth ridges in the external view marked by longitudinal striae, whereas internally there is a prominent adductor ridge. The scutum—tergal segment is narrow. The terga spur furrow is almost closed, and internally, the blue-violet to purple and white parities present a weakly developed crest for the depressor muscle (Fig 2).

**Fig 3:**
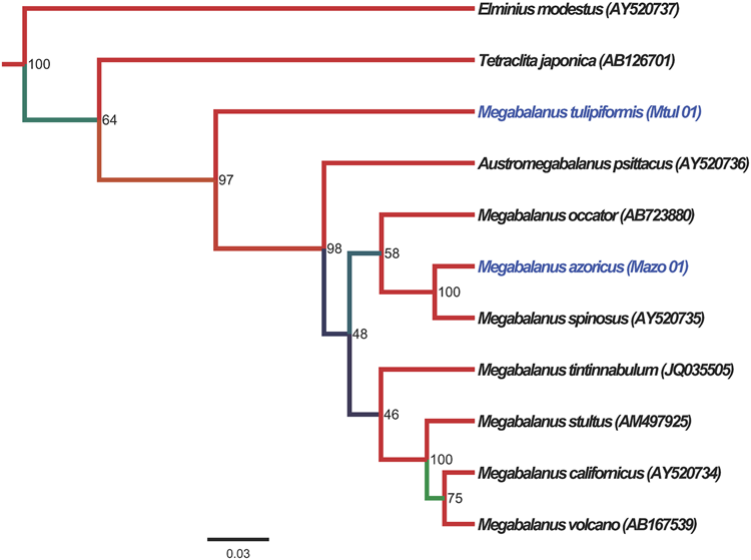
Phylogenetic tree within the *Megabalanus* genus. Phylogenetic tree based on the Bayesian criterion and elaborated with MrBayes from 16SrRNA sequences with distance calculated by the GTR model. *Megabalanus* sequences available from Genbank were included in the analysis, which incorporated 16S rRNA sequences obtained from *M*. *tulipiformis* and *M*. *azoricus* specimens (in blue color). Branch support is indicated by the numbers on branches, reflecting the Bayesian analysis posteriori probabilities in percentages. The *Elminius modestus* sequence was used as an outgroup.

The partial 16S rRNA sequences obtained from the *M*. *azoricus* (Mazo CAN03) and *M*. *tulipiformis* (Mtul 01) specimens, both sampled from the Canary Islands, were 1143 bp and 1192 bp length. The ribosomal alignment consisted of 11 sequences, 484 characters long and comprising 8 sequences from the available partial mitochondrial 16S rRNA gene from diverse *Megabalanus* species (GenBank/EMBL/DDBJ accession numbers: KM575947 and KM575948). This limited phylogenetic inference allowed us to verify the genetic distinctiveness and taxonomic congruence of the sample analyzed, belonging to a species without any available phylogenetically relevant molecular information at present. The *Megabalanus* species (97%) formed a monophyletic group that included *Austromegabalanus psittacus* and *M*. *tulipiformis* in a divergent and basal position. *M*. *azoricus* belonged to a clade that includes its closest relatives *M*. *spinosus*, and basally, *M*. *occator*. The sister clade, which comprises the rest of the species for which 16S rRNA data is available, assembled *M*. *tintinnabulum* in a basal position, but with low support (46%), with respect to the derived clade containing *M*. *stultus* and the closely related *M*. *volcano* and *M*. *californicus* (Fig 3).

Moreover, the publicly available COX1 barcode data from alternative *Megabalanus* taxa, not previously considered in the ribosomal dataset, also suggest its divergence with respect to *M*. *azoricus*. Thus, the *M*. *azoricus* specimen shows a low value of similarity (<86%) with respect to *M*. *zebra* in the BOLD repository and to *M*. *rosa* (JX503004), *M*. *coccopoma*, and *M*. *ajax* records in the Genbank database.

The intraspecific variability, calculated from the COX1 partition data set (*N* = 54, length = 658 bp), was 0.0036 (S.D. = 0.0013), which is in the range of other intraspecific barnacle values: 0.006–0.011 in *Pollicipes* species [24] and 0.022 in *Balanus glandula* [62].

### Genetic diversity

**Fig 4:**
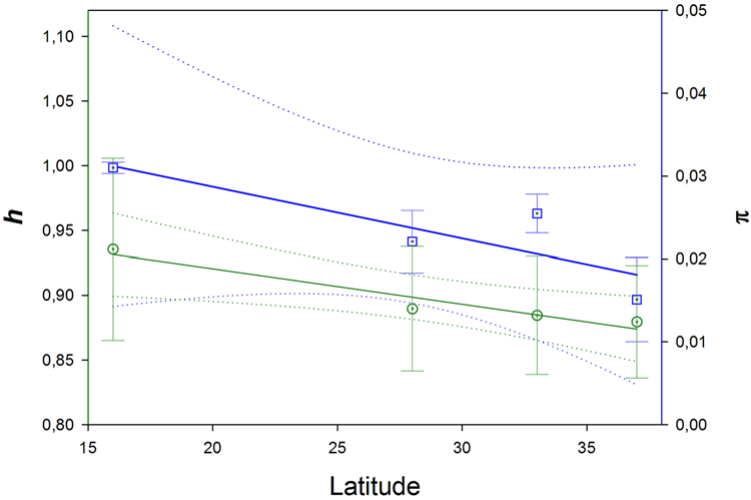
Regression plot of the haplotype diversity and nucleotide diversity. Regression analysis of haplotype diversity (*h*) (blue line) and nucleotide diversity (π) (green line) from mitochondrial control region sequences of the Azorean barnacle (*Megabalanus azoricus*), with respect to sampling site latitudes (Azorean, Madeira, Canary, and Cabo Verde archipelagos).

The population genetic structure of *M*. *azoricus* in Macaronesia was inferred from the complete mitochondrial control region sequences acquired from samples (*N* ≥ 50 individuals) collected along the Macaronesian range, at sites located in the Azores, Madeira, Savage, Canary, and Cabo Verde archipelagos (Table 1, Fig 1). The control region exhibits the expected AT rich (79.35%) and hypervariable profile (24.9% of polymorphic sites). This data set elucidated for population analysis consists of an alignment of 207 sequences, 430 bp length, including 107 polymorphic sites, and 15 gap sites. The control region sequences were deposited in GenBank/EMBL/DDBJ under the accession numbers KM575967-KM576099. All haplotypes from the Cabo Verde sample were private. The nucleotide and haplotype diversity values obtained from the CR in *M*. *azoricus* were moderate. The overall species haplotype diversity (*h*) was 0.9649, being 133 haplotypes with a nucleotide diversity (π) = 0.01813. The population values suggest lower values in the northern Azores population (*h* = 0.8967; π = 0.01242) compared to those of the southern Cabo Verde population (*h* = 0.9984; π = 0.02115) (Table 1). Moreover, the *h* (R^2^ = 0.735, *P* = 0.0022) and π (R^2^ = 0.9035, *P* = 0.0073) slope values were significant according to the latitudinal regression analysis (Fig 4). As is expected for a codifying sequence, the COX1 *h* and π values were lower (*N* = 54, *h* = 0.809, π = 0.00356). These COX1 sequences were deposited in GenBank/EMBL/DDBJ under the accession numbers KM575949—KM575966. The mean intraspecific *p*-distances among COX1 and control region (CR) haplotypes were 0.004 and 0.016, respectively.

In all samples, the θ estimates (4*N*_e_μ) based on segregating sites exceeded those for π, as is expected for expanding populations. Thus, Tajima’s *D* estimate was generally negative although without significant values (*P* > 0.05). Therefore, we could not reject the null model of a neutral genealogy with a constant effective population size, with the exception of the Cabo Verde sample (*P* = 0.012). For the complete set of populations, *D* was negative, being −1.78818 (*P* = 0.005). The Fu’s *Fs* was significant for all samples whereas the *R*_2_ estimators were only significant for the Canary and Cabo Verde samples, with similarly low *Fs* and *R*_2_ values at lower latitudes (Table 1).

### Haplogroup structure

**Fig 5:**
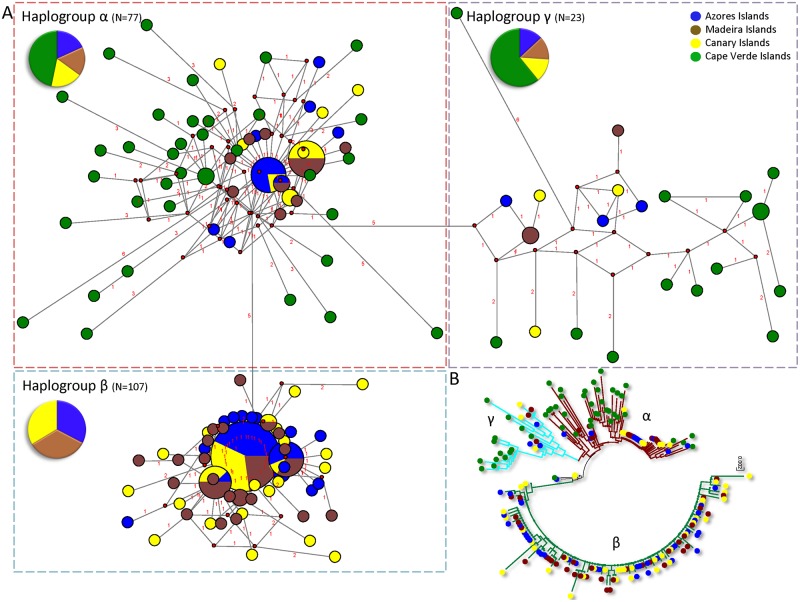
Haplotype network for *Megabalanus azoricus*. A. Haplotype network using a median joining algorithm from the *Megabalanus azoricus* mitochondrial control region DNA sequence alignment. Each code-colored circle represents a single haplotype, whose area is proportional to the haplotype frequency. The length of the connecting lines is proportional to the number of mutational steps. B. Neighbor-joining optimal mid-point rooted tree with the distance measured by the selected Tamura-Nei model, including a rate variation among sites with shape α = 0.21 for the gamma distribution. Branch support was estimated by bootstrapping (2,000 replicates).

The haplotype network revealed three haplogroups (α, β, and γ) disjointed by five mutations. They are equally distributed among the Azores, Madeira, and Canary archipelago samples, whereas in the Cabo Verde sample haplogroup β is absent (Fig 1). The α haplogroup (*N* = 77, *h* = 0.985, π = 0.01073) includes two highly frequent haplotypes. One of them is located mainly in the Azores and Canary Islands; the other occurs, in identical proportion, in the Madeira and Canary Islands. Additionally, the majority of the other divergent and rare haplotypes have been detected in the Cabo Verde sample. The β haplogroup (*N* = 107, *h* = 0.9607, π = 0.004718) is compact, absent from the Cabo Verde, with a most common haplotype (64%), and forms a star conformation with closely related haplotypes. The third haplogroup, γ (*N* = 23, *h* = 0.9921, π = 0.01574), consists of a small number of low frequency haplotypes, mainly from the Cabo Verde sample (Fig 5A). This haplotype relationships and assemblage were also recovered in the neighbor-joining tree where haplogroups were monophyletically clustered and located the α and γ haplogroups in a basal position (Fig 5B). The mean sequence divergence among the haplogroups ranged from 0.019 ± 0.004 to 0.030 ± 0.004, being equidistant haplogroup α, with respect to β and γ. In contrast, mean sequence divergence within haplogroups was only 0.009 ± 0.002. Both Tajima’s *D* and Fu’s *Fs* revealed low, significant (*P* < 0.001) values for the α and β haplogroups as is expected from an expansion event; whereas a non-significant value was observed for haplogroup γ. The *R*_2_ values, being lower values for the α and β haplogroups under the demographic expansion scenario, are congruent with the Tajima’s *D* and Fu’s *F* values (Table 1).

### Population genetic structure

The computed pairwise *F*_*ST*_ were only significant (*P* < 0.001) for the population pairwise comparisons involving the Cabo Verde population with a mean value of 0.366 (S.D. = 0.009). The rest of the populations comparisons were close to 0 and non-significant (Table 2).

**Table 2:** *F*_*ST*_ estimations from pairwise comparisons among the populations of Macaronesian *Megabalanus azoricus* sampled using the mitochondrial control region data.

Population	1	2	3	4
1. Azores		0.9009±0.0333	0.7207±0.0508	0
2. Canary	−0.0129		0.9459±0.0205	0
3. Madeira	−0.0116	−0.0137		0
4. Cabo Verde	0.3650*	0.3580*	0.3758*	

*Significant values *P* < 0.001

*F*_*ST*_ values are below the diagonal whereas *P* values, based in 100 permutations, are above.

In agreement with the *F*_*ST*_ estimations, significant AMOVA values were only observed when the Cabo Verde sample was included in a separate group from the one containing the Azores, Madeira, and Canary Islands. The majority of percentage variation (65.1%) was significantly allocated within populations within the defined groups, resulting in a *Φ*_*ST*_ of 0.35. The percentage variation associated with the separation of the Cabo Verde sample from the other populations had (35.33%) an identical and also significant *Φ*_*CT*_ of 0.35 (Table 3).

**Table 3:** Analysis of molecular variance (AMOVA) based on the mitochondrial control region sequences of the Azorean barnacle, *Megabalanus azoricus*.

Source of variation	Variance components		Variation %	Fixation Indices	*P-value*
Among groups	1.78350	Va	35.33371	Φ_CT_ = 0.35334	0
Among populations within groups	-0.02169	Vb	−0.42972	Φ_SC_ = −0.00665	0.86462
Within populations	3.28578	Vc	65.09601	Φ_ST_ = 0.34904	0
Total	5.38205			

The structure tested included one northern group comprising the Azores, Madeira (including Savage), and Canary sampling sites, and a second group containing only the Cabo Verde sample.

### Demographic history

**Fig 6:**
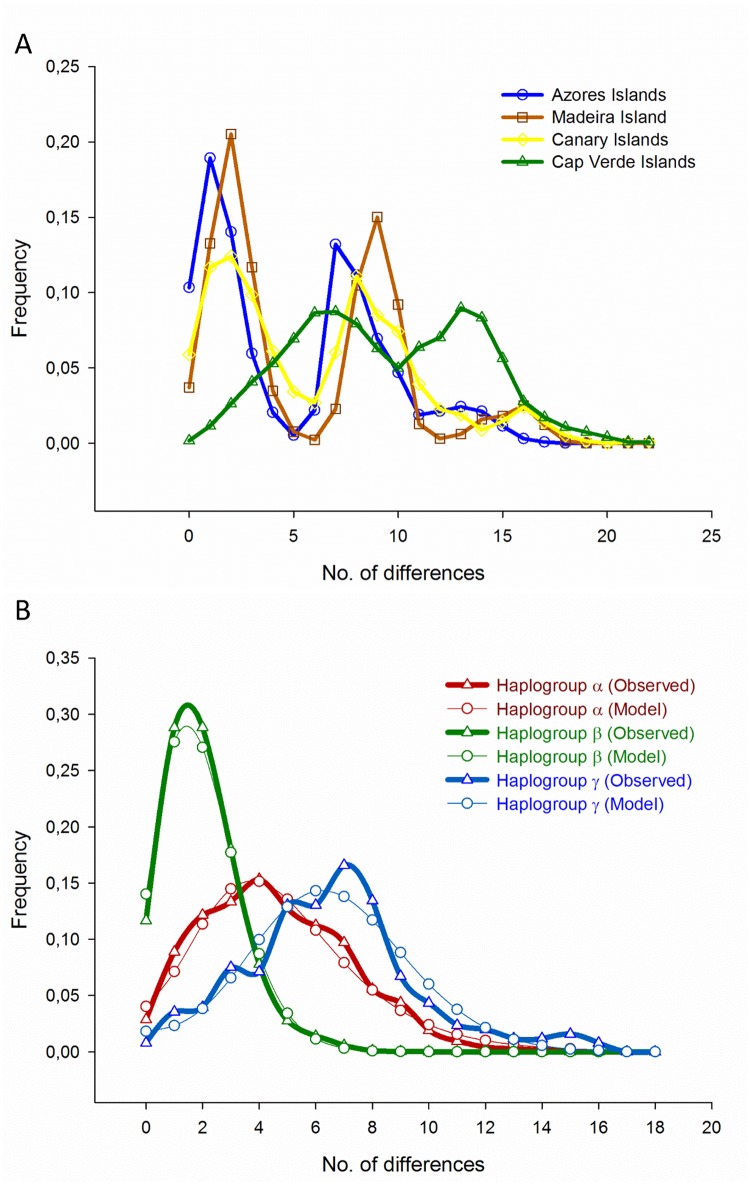
Mismatch distributions for *Megabalanus azoricus*. Mismatch distributions estimated for (A) each of the sample sites in the four Macaronesian archipelagos (Azores, Madeira, Canary, and Cabo Verde Islands) and (B) for the three identified haplogroups distributed along the Macaronesian sampling sites.

The mismatch distributions estimated from the whole species or sampling sites at the four Macaronesian archipelagos are either bi- or multi-modal, reflecting the population data arrangement containing three divergent haplogroups or currently sympatric lineages. The almost superimposed distributions of the Azores, Madeira, and Canary samples clearly differentiated from the right-displaced Cabo Verde bimodal mismatch distribution (Fig 6A). In contrast, the distributions calculated separately for each *M*. *azoricus* haplogroup revealed its historical demography. They were unimodal and consistent with distributions expected for populations undergoing a sudden population expansion (Fig 6B).

The non-significant *SSD* and *Ri* estimates were generally lower in the case of haplogroup distributions, suggesting a better fit with the ones expected for demographic expansion events. The β haplogroup distribution shows a maximum for a small number of differences, reflecting the majority of pairwise comparisons among haplotypes in a star-like conformation. The α and γ haplogroup specific waves were right-displaced, with the highest τ value estimated for the γ distribution (Table 1).

**Fig 7:**
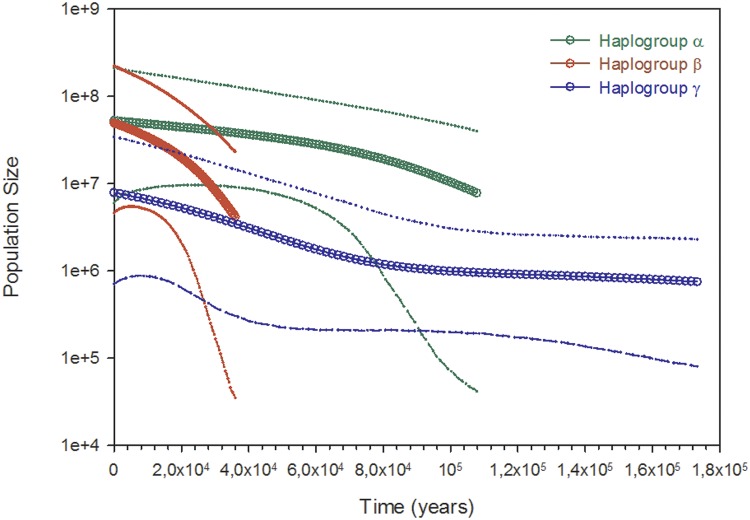
Bayesian skyline plot for *Megabalanus azoricus* haplogroups. Bayesian skyline plot based on mitochondrial control region coalescent analysis for the *Megabalanus azoricus* haplogroups.

The Bayesian coalescent analysis suggested that the oldest haplogroup γ dates back to 180 Kya (thousands years ago) but with a signal of significant growth after 100 Kya. However, this haplogroup has the smallest population size, almost an order of magnitude less than that of haplogroups α and β. The emergence and exponential growth of haplogroup α also occurred around this date (110 Kya). Haplogroup β was dated back to 40 Kya, showing the highest exponential growth to achieve a present population size similar to that of haplogroup α (Fig 7).

In geminate barnacle species (*Euraphia* genus) around the Panamanian Isthmus, a divergence rate of 3.1% My^-1^ (million years) was estimated for the COX1 gene being used for time divergence estimations in *Chthamalus* and *Pollicipes* genera [23, 63, 64]. The present work produced COX1 and control region (CR) comparable data sets for a subsample (*N* = 54). From these data, we estimated the relationship between COX1 and CR gene mutation rates to apply an indirect estimation of the mutation rate to the CR sequences. This approach was based on the estimation of the ratio between each gene-specific *θ* (*θ* = 2*Nμ*, *N* = effective population size, and *μ* = mutation rate for each loci). Because the effective population size (*N*) is identical among linked loci within the same sample, the ratio between *θ*_*COX1*_ and *θ*_CR_ equals the ratio of mutation rates between loci. The *θ* estimates were 3.731 and 8.558 for the COX1 and CR data sets, respectively. Consequently, the control region mutation rate (*μ*_CR_) was 2.294 times the value observed for COX1 gene and, thus, it is estimated as 3.56 x 10^-8^ per site per year. This estimated *μ*_CR_ value was useful for population time estimation using the *t* = *τ*/2*u* formula, *t* being the number of generations since demographic expansion, *τ* the mismatch-distribution parameter value, and *u* the substitution rate per sequence per generation. Considering a generation time of 0.583 years [65], the time since demographic expansion has been estimated as 108,000, 61,000, and 224,000 years for the α, β, and γ haplogroups, respectively. The highest lineage divergence was 4.5% (mean = 2.25%) and considering the 3.56% per million years mutation rate, the oldest haplotype divergence dates back to approximately 632 Kya.

## Discussion

### Taxonomic evaluation

The morphological and molecular species identification carried out was necessary to confirm the taxonomic congruence within the analyzed sample set, considering i) the geographically isolated sample sites, ii) the complex taxonomy, with incomplete descriptions, major revisions, and a number of synonymies [2, 9]; iii) the invasive ecology of these taxa [10, 66], and iv) the presence of morphologically related Macaronesian sympatric taxa and forms, with unknown relationships among them [9]. As a result, our Azorean barnacle, *Megabalanus azoricus*, sample sets have been defined as a well-supported species based on diagnostic morphological characters and molecular data, confirming its taxonomic consistency throughout the Macaronesian region.

The taxonomic assignation of the samples analyzed was based on morphological characterization [2, 8], the sampling from the *M*. *azoricus* type locality (Terceira, Azores Islands), and their monophyly with relatively low level of intraspecific genetic variability. Comparison of our mitochondrial sequences with the homologous data available for other *Megabalanus* species, *M*. *tulipiformis*, *M*. *occator*, *M*. *spinosus*, *M*. *stultus*, *M*. *californicus*, *M*. *tintinnabulum*, *M*. *volcano*, *M*. *zebra*, *M*. *rosa*, *M*. *coccopoma*, and *M*. *ajax*, suggest the distinctiveness and taxonomic singularity of the present taxa. Moreover, among the species not considered above and without available genetic information only *M*. *vesiculosus*, with distinctive characters (e.g. spinose ribs), has an Atlantic distribution, although it is apparently extinct from the Brazilian coast. Among the species analyzed in the 16S rRNA data set, *M*. *azoricus* was closely related to *M*. *spinosus*, which shows a southern distribution across the Guinean Gulf, the Principe, Sao Tomé, and Annobon islands [2], suggesting a tropical ancestry for *M*. *azoricus*. The phylogenetically divergent *M*. *tulipiformis*, with shared distribution in the East Atlantic although absent in the Azores [9], also shows clearly differentiated characters (e.g. equal orifice and basal diameter) and has a deeper bathymetric range than the intertidal *M*. *azoricus* [2, 13].

Despite the limited ribosomal sequence data, both in taxa and sequence length, the present *Megabalanus* data supplements the extensive barnacle phylogeny inferred in other studies [16, 19], which do not include *M*. *azoricus*, *M tulipiformis*, and other *Megabalanus* species considered here. These phylogenies included independent molecular and fossil calibrations that support the validity of our COX1 calibration. Thus, *Austromegabalanus victoriensis* [67] has been used to calibrate speciation in the *Megabalanus* genus, which has been dated to 11.6–23 Mya. The highest *Megabalanus* interspecific distance, among the available COX1 sequences, was 0.215 when comparing the pairwise *M*. *coccopoma* and *M*. *ajax*. Using the 1.55%/My mutation rate a minimum speciation time for the origin of the *Megabalanus* genus can be roughly dated to ~14 Mya, which agrees with barnacle divergence time estimations based on fossil calibration [18].

### Genetic diversity and population demography

The haplogroup pattern of ancestral lineages retained after a lineage sorting process falls within the category II in Avise’s classification of phylogeographic patterns. This category is characterized by the presence of highly divergent and broadly sympatric lineages, theoretically occurring in species with a large evolutionary *N*_*e*_ and abundant gene flow [68]. Nevertheless, there is a disparity within haplogroup-specific π values, as also revealed by the mismatch distributions, with the lowest value estimated for haplogroup β. Likewise, the overall species haplotype diversity (*h*) (0.9649) differs from the higher *h* value estimated for haplogroup γ (0.9921). The maximum values from haplogroup γ agree with the expected estimates for a population characterized by a stable, large and long-term *N*_e_ [68]. In the case of haplogroup β, the low haplotype and nucleotide diversity are consistent with a recent demographic expansion, spatially circumscribed to northern archipelagos, with a single majoritarian haplotype and closely related haplotypes in a star-like structure.

Both π and *h* values at the species level (π = 0.0181, *h* = 0.9649) were lower than those estimated for other Cirripedia species. Considering the homologous control region sequence, the stalked barnacle, *Pollicipes pollicipes*, had a π = 0.0330 and *h* = 0.9936 [23]. Also in *Semibalanus balanoides*, π = 0.0298 and *h* = 0.9967 were estimated from a European sample (UK, *N* = 43) [26]. Similarly, the π from COX1 (0.0036) is below the mean calculated for Cirripedia species (0.0093) [69]. Higher π and *h* COX1 values are obtained in other species such as *Tetraclita rubescens* (π = 0.0094, *h* = 0.9998), which also included the control region [27] and *Chthamalus malayensis* (π = 0.005–0.017 and *h* = 0.95–1.00) [25]. Furthermore, estimated values are slightly higher for the Japanese populations of congeneric *M*. *volcano* (π = 0.0099, *h* = 0.847) and *M*. *coccopoma* (π = 0.00701, *h* = 0.906) species, but were similar to those for *M*. *rosa* (π = 0.00386, *h* = 0.819) [10]. Repeated demographic bottleneck events and a relatively small population size are likely responsible for the low π and *h* observed in this insular endemic species [68] when compared to species that have a larger distribution range.

The negative relationship between latitude and genetic diversity (*h* and π) observed is a recurrently described pattern, also observed in diverse crustacean species [23, 70] and other marine invertebrates along this latitudinal range [71]. A parallelism with respect to the species richness diversity patterns at tropical latitudes [15], promoted by diverse debated mechanisms, such as larval dispersal capacity [72], life history, demography, population structure, rate of molecular evolution, and rate of speciation has been suggested [73]. However, at the intraspecific level, the above cited variables are fixed and the process responsible for this pattern is frequently associated with post-glacial range expansion from southern refugia, and likely relies on low lineage extinction rates at low latitudes [74].

All of the Tajima’s *D* population values were negative but also non-significant, with the exception of the Cabo Verde sample. Significant *D* values were only estimated for the α and β haplogroups, suggesting a recent expansion. The non-significant *Megabalanus azoricus D* value (−1.15875, *P*>0.1) was similar to the mean *D* value (−1.087) estimated from Cirripedia COX1 data [69]. However, this mean value is above the significant *D* estimation for the species (−1.7882; *P*<0.01) for haplogroups α and β (−2.46126; *P*<0.01 and −2.64192; *P*<0.001; respectively) when using the non-codifying control region sequence. Although a recurrent negative skewness in the empirical distribution of *D* statistic values for Cirripedia [69] has been described, the present estimations seem to recover a true expansion dynamics signal. Accordingly, in *M*. *azoricus*, negative, significant *D* values are not universal, thus haplogroup *γ* shows a non-significant *D* value as expected for a long standing stable lineage in agreement with inferences from sequence diversity, mismatch distributions, and dating estimates. Moreover, we could not reject the null hypothesis based on the Fu’s *Fs* statistic of constant size for the haplogroup γ, whereas that of the *R*_2_, was a higher, although significant value (*P* = 0.026) for this haplogroup (Table 1).

The genetic signature of putative demographic bottlenecks associated with extinction and re-colonization dynamics in northern archipelagos contrast with long-term stable population signals in the low latitude Cabo Verde population. Thus, the low number and closely related haplotypes, particularly in the Azores, reflect the genetic diversity erosion in northern latitudes by means of low-frequency haplotype losses, re-colonization associated founder events, and the persistence of common haplotypes. In contrast, the high diversity values in Cabo Verde reflect the long-term persistence of lineages in an evolutionarily stable environment.

### Population Structure

The *F*_*ST*_ and AMOVA analysis of the geographic partition of genetic diversity suggested two opposing patterns. First, a homogeneous gene pool comprises the northern archipelagos of the Azores, Madeira, Savage, and Canary Islands, which exhibit identical distributions of the three haplogroups detected and with the most recent haplogroup β (52%) comprising the majority. Xavier et al. [71] observed a similarly homogeneous pool at these same locations for the sponge, *Phorbas fictius*, except for the Madeira differentiated population. Second, an isolated southern population in the Cabo Verde Islands, where genetic diversity is high but haplogroup β is absent. Gene flow between these two population sets is low enough to promote the significant genetic isolation observed between northern and southern populations. The northern populations are subject to common oceanographic and paleoclimatic regimes. These archipelagos are under the influence of the Azores and Canary Currents, they have a similar geological origin including historically emerged intermediate seamounts available as stepping-stones during colonization [28] and a lot of human-mediated maritime connections [75]. Consequently, shared historical and actual homogenizing processes are likely responsible for the common pattern observed. In contrast, the Cabo Verde archipelago is under a different climate and oceanographic regime with a composite biogeographic character, having both Caribbean and Atlantic-Mediterranean affinities regarding the marine biota [14]. As a result, this biogeographic divergence is to varying degrees reflected in genetic discontinuities observed in related and other marine invertebrates [23, 24, 76]. The observed isolation of Cabo Verde is supported by favorable contemporary hydrographic dynamics influencing planktotrophic larval dispersion [23]. At the northern branch of the subtropical gyre, the Azores current transports North Atlantic Central Water (NACW) eastward. The southeasternmost branch originates the Canary Current (CC), which flows from 33° N to 20–25° N and southward along the African coast. However, it is prevented from reaching Cabo Verde at the Cabo Verde Frontal Zone (CVFZ), which is characterized at the northern limit by the predominance of NACW water, whereas waters south of the CVFZ resemble equatorial conditions [77]. The southwestern flow drives CC waters to the open ocean where it feeds the North Equatorial Current. This hydrographic discontinuity generated by the CVFZ agrees with the genetic divergence observed in populations on both sides of the CVFZ [23]. Therefore, the front presents a crucial physical component of a biologically effective barrier for larval dispersion. Additionally, recruitment is hindered in insular habitats by the intense upwelling systems induced by the southwestward trade winds in the region between Cape Blanc and the Canary Islands [78].

In contrast, mesoscale hydrographic structures play a key role in the settlement and recruitment of other crustacean species [23, 79], particularly in the critical littoral zone. For example, the intense mesoscale events and the island mass effect in the Azores [6], Madeira [80], Canary [81, 82], and Cabo Verde Islands [83].

### Dating and process for haplogroup pattern

Pleistocene climate changes with the associated sea surface temperature (SST) and sea-level fluctuations are a paradigm in marine biogeography and sustain the current phylogeographic hypothesis. Both variables affect the highly sensitive rocky intertidal habitat, particularly for the sessile *M*. *azoricus*. During maximum glacial episodes the glacial front reached as far south as 40° N [84] and is associated with negative consequences for the warm fauna of the Azores archipelago [85]. However, the effect of SST variation on the southernmost archipelagos, particularly Cabo Verde, is probably negligible. Additionally, fluctuations in sea-level alter habitat availability with respect to seamounts [28].

The small populations inhabiting isolated insular habitats and/or glacial refugia fuel population differentiation [37]. The intensification of the CCA, NEC, and coastal upwelling at the southernmost Macaronesian refuges are conditions favoring insular isolation [86]. Less astringent current patterns and intermediary and dispersed seamounts operating as stepping-stones [28, 76] likely supported a limited northward colonization process.

Within the Macaronesian marine region, it is noteworthy that the paleoclimatic records from ODP Site 658, off Mauritania, agree with the abundant paleo-oceanographic data. Both ln (Si/Al) and records of percentage sedimentary opal reflect the deglacial events, allowing an accurate dating of glacial termination II and the start of interglacial period MIS 5 to 130 Kya, whereas termination III and MIS 7 interglacial occurred 240 Kya [87]. The MIS3 epoch took place between 60 and 24 Kya [88]. The widely distributed haplogroups with the highest polymorphism level (α and γ) are expected to be older than haplogroup β. Moreover, the mismatch distributions, Bayesian coalescent analysis, and the basal position of the α and γ haplogroups within the mid-point rooted phylogenetic tree support this relatively broad temporal dating. Thus, the estimated expansion dates of the ubiquitous α and γ haplogroups place these events within the MIS 5 and MIS 7 interglacial periods, respectively. Similarly, the β haplogroup dating preceded the Last Glacial Maximum, putting it in the MIS 3 interglacial. Coincidentally, demographic expansion of European and African stalked barnacle, *Pollicipes pollicipes*, populations date back to the same paleoclimatic period 87–118 Kya, with the most recent common ancestor dated between 200 and 170 Kya [23, 64].

Cabo Verde has well-established refugia and ancestral area features [89], as suggested by the close phylogenetic relationships between *M*. *azoricus* and the eastern Atlantic tropical *M*. *spinosus*. The higher diversity values along a latitudinal gradient for both the α and γ haplogroups suggest that these lineages originated in the stable refuge habitat of the Cabo Verde islands. Additionally, haplogroup α possesses a limited number (2) of highly frequent haplotypes in contrast to a number of private haplotypes from Cabo Verde, as is expected from few founder events or genetic diversity erosion. In contrast, the most recent haplogroup, β (40–61 Kya), is distributed exclusively throughout northern locations (Canaries, Savage, Madeira, and Azores) suggesting the persistence of an effective oceanographic barrier to gene flow, as detailed above, from MIS3, selection against Cabo Verde subtropical habitat, and/or a strong contemporary human-mediated northern dispersion. This dispersion is sustained by a high level of marine connectivity through fouling on vessels circulating among the northern archipelagos, which is significantly less in Cabo Verde. This human-mediated vagility is responsible for the cosmopolitan distribution of the congeneric species *M*. *coccopoma*, *M*. *rosa*, and *M*. *volcano* [10] and other Balanidae colonization events in Macaronesia [90]. However, the absence of any shared haplotypes between the Cabo Verde population and the other northern archipelagos agrees with the long-term barrier hypothesis, as suggested by the persistence of the Azores front from interglacial and LGM times [91].

A similar colonization pattern was suggested for another Macaronesian endemic, *Tectarius striatus* [76, 92]. Here, Cabo Verde is also considered the ancestral area, with dispersion mediated by counterclockwise currents and available seamounts, and, in contrast, a recently established gene flow barrier between Cabo Verde and the northern archipelagos.

### Macaronesian endemism in two biogeographic provinces

The level of local endemism is highly variable throughout Macaronesia. Cabo Verde and the Azores have the lowest species richness and endemism ranking for oceanic islands, in terms of native higher plant species [35]. The biogeographic data shows a low level of Azorean marine endemism [85, 93–95]. Particularly concerning intertidal space occupiers, marine animal diversity and endemism are lower in the Azores compared to the Canaries and Madeira [95]. The stalked barnacle *P*. *pollicipes* and *C*. *montagui* are absent from the Azorean intertidal, whereas they are recorded from other Macaronesian islands. Similarly, other intertidal taxa such as mussels, patellid limpets, and large trochids are absent or barely represented, likely because of limited food availability for filter feeders [95].

Until now endemic character and evolutionary origin hypotheses concerning *M*. *azoricus* were obscured by the lack of information regarding the relationships between the Azorean type and specimens from the rest of Macaronesia [95]. Thus, the present data support the hypothesis of a widely distributed endemism throughout Macaronesia, with an evolutionary origin in the Cabo Verde Islands, and a historical colonization of the Canaries and Madeira (both are considered the biodiversity nuclei of this region based on the observed endemism levels), and the Azores.

The Macaronesia *sensu lato*, including Cabo Verde, spans two classic biogeographic provinces, Lusitanian and West African, with their common boundary located in the Cabo Verde Islands [94]. Around central Macaronesia, with accepted zoogeographical affinities, differences in dispersal potential confers varying affinity degrees among close regions. In contrast, distant islands with low affinities, the Azores and particularly Cabo Verde, lack a dispersal support [35]. Congruently, the geographic affinity of the well characterized ichthyofauna shows a primary clustering of the Azores, Madeira, and the Canaries, whereas the highest divergence is observed in Cabo Verde [6]. However, *Ophioblennius atlanticus* from the Azores and Cabo Verde cluster in a divergent Lusitanian clade differentiated from the West-African province populations [96].

The concept of a Macaronesian biogeographic entity has been challenged because of its flora, for example, the divergent phytogeographic affinities of the Cabo Verde pteriodophytes [29]. Present data and other phylogeographic analyses of marine invertebrates suggest a similar divergent pattern [23, 24, 76, 92] modified by life cycle, vagility, behavior, and ecology. Further study is necessary to evaluate the phylogeographic hypothesis on a generalized marine pattern defined by the Cabo Verde divergence within a Macaronesian *sensu lato* biogeographic unit or with respect to the Macaronesian *sensu stricto*, originating from and sustained by paleoclimatology and oceanography, respectively. Any effort in pattern definition is crucial in guaranteeing the conservation of genetic diversity and the sustainability of marine resources, particularly for those species that are heavily exploited, as is the case for *M*. *azoricus*.
